# Effects of an online caregiver respiratory education program on sleep-related outcomes and parental stress in children with neuromuscular diseases: a randomized controlled trial

**DOI:** 10.1186/s12887-026-07465-3

**Published:** 2026-08-05

**Authors:** Malihe Sadat Moayed, Sajjad Khaksar, Mehdi Jafari Oori, Forogh Sarhangi

**Affiliations:** 1 Nursing Care Research Center, Clinical Sciences Institute, Faculty of Nursing, Baqiyatallah University of Medical Sciences, Tehran, Iran; 2 Student Research Committee, Baqiyatallah University of Medical Sciences, Tehran, Iran

**Keywords:** Neuromuscular Diseases, Parental Stress, Quality of Life, Respiratory Therapy, Sleep Disorders

## Abstract

**Background:**

Children with neuromuscular diseases (NMDs) commonly experience sleep disturbances related to respiratory dysfunction, which may also increase caregiver stress.

**Objective:**

This study evaluated the effects of a structured caregiver respiratory education program on sleep-related outcomes in children with NMDs and parental stress.

**Methods:**

A randomized controlled pretest–posttest trial was conducted between December 2023 and April 2024 in Tehran and Isfahan, Iran. Ninety-nine children with NMD and their primary caregivers were randomly assigned to an intervention group (*n* = 50) or a control group (*n* = 49). The intervention group received four 75-minute online educational sessions over two weeks focusing on respiratory care techniques, including diaphragmatic breathing, airway clearance, postural drainage, effective coughing and suctioning, positioning, and basic cardiopulmonary resuscitation. Attendance was monitored during sessions. The control group received routine hospital-based education. Outcomes were assessed at baseline and two weeks post-intervention using the Children’s Sleep Habits Questionnaire (CSHQ) and Stress Response Inventory (SRI).

**Results:**

No significant between-group differences were observed in the change in total sleep quality (between-group difference = − 0.12, 95% CI: −1.46 to 1.23; *P* = .286) or parental stress scores (between-group difference = − 0.89, 95% CI: −2.93 to 1.14; *P* = .871). However, exploratory analyses showed significant improvements in bedtime resistance (*P* = .01), sleep anxiety (*P* < .001), and parasomnias (*P* = .01) in the intervention group compared with the control group. Somatization scores also differed significantly between groups (*P* = .02), whereas no significant differences were observed for the remaining parental stress domains. Given the multiple subscale comparisons performed, these findings should be considered exploratory and interpreted with caution.

**Conclusion:**

Caregiver respiratory education may improve selected sleep-related behaviors in children with neuromuscular diseases and may have beneficial effects on specific dimensions of caregiver stress. Although no significant effects were observed on overall sleep quality or total parental stress, caregiver respiratory education may serve as a complementary rehabilitation approach for families of children with neuromuscular diseases. Further studies with longer follow-up periods and objective sleep outcome measures are needed to confirm these exploratory findings.

**Trial registration:**

Iranian Registry of Clinical Trials (IRCT; IRCT20221231056998N1) registered on April 25, 2023.

## Introduction

Neuromuscular diseases (NMDs) in children comprise a heterogeneous group of inherited and acquired disorders characterized by progressive muscle weakness, impaired mobility, and respiratory dysfunction [1, 2]. Conditions such as Duchenne muscular dystrophy and spinal muscular atrophy are commonly associated with respiratory muscle weakness, ineffective airway clearance, and nocturnal hypoventilation, all of which may contribute to disrupted sleep patterns and reduced sleep quality in affected children [3]. Sleep disturbances in children with NMDs have been associated with daytime fatigue, impaired functioning, and decreased quality of life [4, 5].

The chronic and progressive nature of NMDs also places a considerable psychological and physical burden on caregivers. Parents of children with NMD frequently experience persistent stress, anxiety, sleep disruption, and emotional exhaustion related to continuous caregiving responsibilities and concerns regarding respiratory complications [6, 7]. Elevated caregiver stress may adversely affect family functioning, caregiving capacity, and ultimately child health outcomes [8, 9].

Current management strategies for pediatric NMDs increasingly emphasize multidisciplinary and family-centered approaches, particularly in the context of long-term respiratory care. Previous studies suggest that caregiver education programs focused on respiratory management and home-based supportive care may improve treatment adherence, caregiver confidence, and selected health outcomes in children with chronic respiratory or neuromuscular conditions [10–12]. Interventions targeting airway clearance, positioning, and respiratory symptom management may also help reduce nighttime discomfort and improve sleep-related functioning [13].

Despite these potential benefits, evidence regarding the effectiveness of caregiver-focused respiratory education programs in children with NMDs remains limited, particularly in randomized controlled settings. In addition, few studies have simultaneously examined child sleep outcomes and caregiver psychological responses following respiratory care education [14, 15].

Therefore, this randomized controlled trial aimed to evaluate the effects of a structured respiratory care education program for parents of children with NMDs on children’s sleep quality and parental stress. We hypothesized that caregiver-focused respiratory education might improve selected aspects of sleep-related functioning and support caregiver well-being as an adjunct to standard pediatric rehabilitation care.

## Methods

### Study design and participants

This randomized controlled pretest–posttest trial was conducted between December 2023 and April 2024 in pediatric neuromuscular rehabilitation clinics affiliated with medical centers in Tehran and Isfahan, Iran. The study was approved by the institutional ethics committee, and written informed consent was obtained from all participating parents or legal guardians prior to enrollment.

**Fig. 1:**
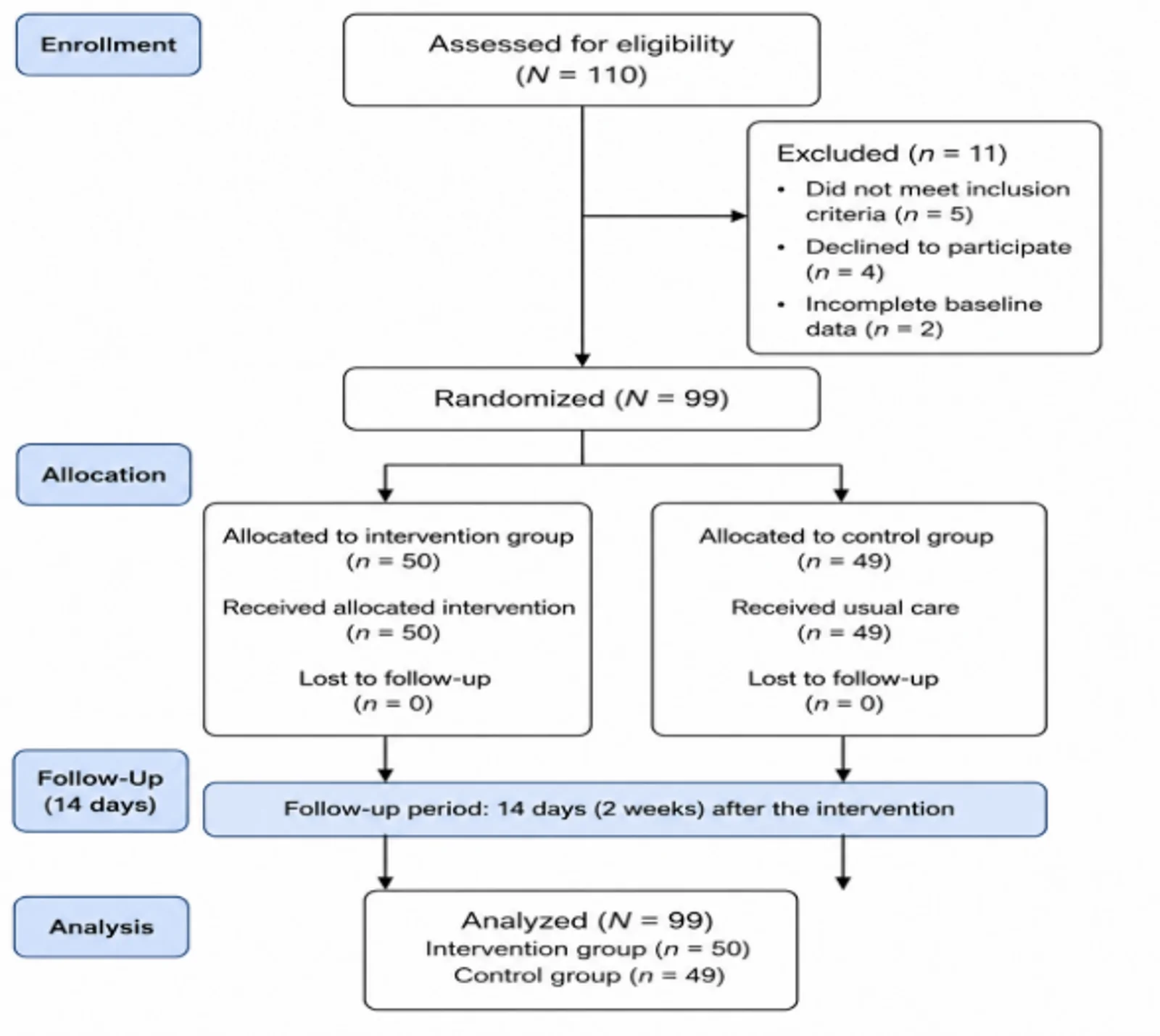
CONSORT Flow Diagram

Participants were recruited from a previously conducted descriptive study involving 207 children with neuromuscular diseases (NMDs) and their parents attending pediatric neuromuscular rehabilitation clinics [9]. During the descriptive phase, parents completed the Persian version of the Children’s Sleep Habits Questionnaire (CSHQ). Children with a total CSHQ score ≥ 41, indicating clinically significant sleep disturbance, were considered eligible for the interventional phase. All eligible families were invited to participate, and those who met the inclusion criteria and provided written informed consent were enrolled in the trial (Fig. 1). Eligible participants included children aged 4–14 years with a confirmed diagnosis of a neuromuscular disease (NMD), including spinal muscular atrophy (SMA), Duchenne muscular dystrophy (DMD), Becker muscular dystrophy (BMD), and other clinically confirmed neuromuscular diseases. Diagnoses were established by treating pediatric neurologists and confirmed through the participants’ medical records. Each child had a parent willing to participate in the online educational sessions. Exclusion criteria included severe cognitive impairment preventing questionnaire completion, unstable acute medical conditions requiring hospitalization during the study period, or the inability of parents to participate in the online educational sessions.

### Sample size, randomization, and allocation concealment

Sample size was calculated a priori using G*Power software (version 3.1.9.7) based on the study’s primary outcome, parental stress. The required sample size was estimated using data from a pilot study conducted prior to the main trial, in which the mean parental stress score after the intervention was 80 (SD = 4.5) in the intervention group and 83 (SD = 5.0) in the control group, corresponding to a Cohen’s d effect size of approximately 0.63. Using an independent-samples t test (two-tailed), a significance level of 0.05, a statistical power of 80% (1 − β = 0.80), and an allocation ratio of 1:1, the required sample size was estimated to be 40 participants per group. To account for an anticipated 10% attrition rate, the target sample size was increased to 44 participants per group.

Following baseline assessment, eligible participants were randomly assigned to the intervention or control group in a 1:1 ratio using Random Allocation Software with permuted blocks of four. Allocation concealment was ensured using sequentially numbered, opaque, sealed envelopes prepared by an independent researcher who was not involved in participant recruitment, intervention delivery, or outcome assessment. Outcome assessors and data analysts were blinded to group allocation throughout the study.

### Intervention

The educational program was developed by the research team based on current evidence and clinical guidelines for the respiratory care of children with neuromuscular diseases. Before implementation, the educational content was reviewed by a panel of experts in pediatric nursing, pediatric neurology, and respiratory rehabilitation to establish its content validity. Following expert feedback, the educational materials were revised accordingly prior to delivery. Parents in the intervention group participated in a structured respiratory care education program consisting of four 75-minute live online webinars delivered over a two-week period (two sessions per week). Educational content focused on practical respiratory care strategies commonly used in home management of children with NMDs and included diaphragmatic breathing exercises, airway clearance and postural drainage techniques, effective coughing and suctioning methods, positioning strategies, and basic cardiopulmonary resuscitation (CPR). To reinforce learning, supplementary educational materials including presentation slides, instructional videos, audio guides, and PDF handouts were provided after each session. Throughout the intervention period, parents were able to contact the research team to ask questions or seek clarification regarding respiratory care techniques. They were also instructed to report any physical or psychological adverse events potentially related to the intervention throughout the study period.

Intervention fidelity was monitored through webinar attendance logs and interactive participation during the sessions, including real-time discussion and question-and-answer exchanges. The control group received routine hospital-based education typically provided to families of children with NMDs. Following completion of the study, all educational materials were made available to caregivers in the control group.

### Outcome measures

Children’s sleep quality was assessed using the Persian version of the Children’s Sleep Habits Questionnaire (CSHQ), a validated parent-reported instrument that evaluates multiple domains of pediatric sleep behavior, including bedtime resistance, sleep anxiety, parasomnias, and sleep-disordered breathing. The CSHQ consists of 35 items, of which 33 scored items contribute to the total score across eight domains. Items are rated on a three-point Likert scale (“usually”, “sometimes”, and “rarely”), yielding a total score ranging from 33 to 99, with higher scores indicating poorer sleep quality. A total score of ≥ 41 was considered indicative of clinically significant sleep disturbance [16].

Parental stress was assessed using the Persian version of the Stress Response Inventory (SRI), a validated self-report instrument that evaluates emotional, physical, cognitive, and behavioral responses to stress across seven domains: tension, aggression, somatization, anger, depression, fatigue, and frustration. The questionnaire consists of 39 items rated on a five-point Likert scale ranging from 0 (“not at all”) to 4 (“completely”). The total score ranges from 0 to 156, with higher scores indicating greater levels of parental stress [17]. Validated Persian versions of instruments were used in this study. The Persian version of the Children’s Sleep Habits Questionnaire (CSHQ) has demonstrated acceptable validity and reliability, with a Cronbach’s α of 0.82 [18]. Similarly, the Persian version of the Stress Response Inventory (SRI) has been shown to have excellent validity and reliability, with a Cronbach’s α of 0.94 [19]. Outcome measures were completed by parents at baseline before randomization and again two weeks after completion of the intervention.

### Statistical analysis

Data were analyzed using SPSS version 21 (IBM Corp., Armonk, NY, USA). Descriptive statistics were used to summarize demographic and clinical characteristics. Normality of continuous variables was assessed using the Shapiro-Wilk test. Because several outcome variables demonstrated non-normal distributions, nonparametric statistical tests were used.

Within-group comparisons between pre- and post-intervention scores were performed using the Wilcoxon signed-rank test, and between-group comparisons were conducted using the Mann-Whitney U test. A P value of less than 0.05 was considered statistically significant. Analyses of sleep and stress subscales were considered exploratory and interpreted cautiously because of the potential for type I error associated with multiple comparisons. All randomized participants completed follow-up assessments and were included in the final analysis.

## Results

### Participant flow and baseline characteristics

A total of 110 families were assessed for eligibility, of whom 99 met the inclusion criteria and were randomized to the intervention group (*n* = 50) or control group (*n* = 49)(Fig. 1). All randomized participants completed the study and were included in the final analysis. No adverse events related to the intervention were reported .

The mean age of children was 8.05 ± 2.50 years in the intervention group and 8.60 ± 2.80 years in the control group (*P* = .38). Most demographic and socioeconomic variables were comparable between groups. However, a statistically significant difference in gender distribution was observed, with a higher proportion of female children in the intervention group (30/50, 60.0%) compared with the control group (17/49, 34.7%; *P* = .009). Disease subtype distribution was generally comparable between groups (Table 1).

**Table 1:** Comparison of demographic variables in intervention and control groups

Variable	Intervention Group (*n* = 50)	Control Group (*n* = 49)	Test Statistic	*P* value
Age of Child (years)	8.05 ± 2.5	8.6 ± 2.8	t(97) = -1.03	0.38
Gender			χ²(1) = 6.78	0.009
Male	20 (40%)	32 (66%)		
Female	30 (60%)	17 (34%)		
Number of children in family, Median (IQR)	2.0 (1.0–2.0)	1.0 (1.0–2.0)	Z = -1.20	0.23
Father’s Education			χ²(2) = 0.30	0.86
High School or Less	34 (69%)	35 (71%)		
Bachelor’s Degree	11 (21%)	10 (20%)		
Postgraduate	5 (10%)	4 (8%)		
Mother’s Education			χ²(1) = 1.53	0.22
High School or Less	37(73%)	35(71%)		
Bachelor’s Degree	13(27%)	14(29%)		
Use of specific medication			χ²(1) = 0.22	0.64
Use	27(56%)	29(60%)		
Non-use	23(44%)	20(40%)		
Type of disease			χ²(3) = 2.65	0.45
SMA1	11(23%)	4(8%)		
SMA2	20(44%)	29(62%)		
SMA3	15(31%)	12(25%)		
Other NMD	4(8%)	4(8%)		

*SMA: spinal muscular atrophy; NMD: neuromuscular disease; SD: standard deviation; IQR: interquartile range

**†**Due to zero frequencies in the postgraduate category for mothers, bachelor’s degree and postgraduate degree were combined

‡Fisher’s exact test was used due to small expected cell frequencies in the SMA type 1 category (expected count < 5).*Significant baseline difference in gender distribution was observed (*P*=.009). However, all outcome analyses were adjusted for gender, and the results remained unchanged.*

Most parents had a high school education or lower, and the majority of mothers were homemakers. More than half of the participating families reported poor economic status. No significant between-group differences were observed in employment status, insurance coverage, or housing characteristics (Table 2).

**Table 2:** Socioeconomic and family characteristics in intervention and control groups

Variable	Intervention Group (*n* = 50)	Control Group (*n* = 49)	Test statistic	*P* value
Economic Status(n%)			χ²=2.16	0.34
Good	3 (6.0%)	5 (10.2%)		
Moderate	20 (40.0%)	15 (30.6%)		
Poor	27 (54.0%)	29 (59.2%)		
Father’s Employment (n%)			χ²=4.07	0.38
Full-time	17 (34.0%)	15 (30.6%)		
Part-time	19 (38.0%)	16 (32.7%)		
Unemployed	6 (12.0%)	12 (24.5%)		
Self-employed	8 (16.0%)	6 (12.2%)		
Mother’s Employment(n%)			Fisher’s exact	0.27
Full-time	1 (2.0%)	2 (4.1%)		
Part-time	2 (4.0%)	3 (6.1%)		
Homemaker	45 (90.0%)	40 (81.6%)		
Unemployed	2 (4.0%)	4 (8.2%)		
Health Insurance Coverage(n%)			Fisher’s exact	0.87
Yes	47 (94.0%)	47 (95.9%)		
-No	3 (6.0%)	2 (4.1%)		
Housing Status(n%)			χ²=6.48	0.09
Owner	24 (48.0%)	20 (40.8%)		
Tenant	16 (32.0%)	21 (42.9%)		
Living with Family	9 (18.0%)	8 (16.3%)		
No Permanent Residence	1 (2.0%)	0 (0.0%)		

### Outcomes: sleep quality and parental stress

At baseline, no significant differences were observed between the intervention and control groups in total sleep quality scores (Z = − 0.77, *P* = .43) or total parental stress scores (Z = − 0.86, *P* = .38), indicating comparable baseline characteristics for both primary outcomes.

The between-group difference in change in sleep quality was − 0.12 points (95% CI: −1.46 to 1.23), with no statistically significant difference between groups (Mann–Whitney U = 1071.5, *P* = .286). Likewise, the between-group difference in change in parental stress was − 0.89 points (95% CI: −2.93 to 1.14) and was not statistically significant (Mann–Whitney U = 1201.0, *P* = .871)(Table 3).

**Table 3:** Pre- and post-intervention outcomes, change scores, and within- and between-group comparisons for sleep quality and parental stress

Variable	Group(*n*)	Pre-Intervention (Mean ± SD)	Post-Intervention (Mean ± SD)	Change (Mean ± SD)	Between-group difference in change (95% CI)	Between-group Comparison of Change Scores (Mann–Whitney U Test)	Within-group Comparison (Wilcoxon Signed-Rank Test)
Sleep Quality	Intervention(50)	77.66 ± 8.62	76.90 ± 8.48	−0.76 ± 2.63	−0.12 (− 1.46 to 1.23)	U = 1071.5, Z = − 1.07, *P* = .286	Z = − 1.72, *P* = .08
	Control(49)	76.91 ± 8.21	76.27 ± 8.67	−0.65 ± 3.93			Z = − 1.41, *P* = .15
Parental Stress	Intervention(50)	101.27 ± 31.88	102.50 ± 31.80	1.29 ± 2.41	−0.89 (− 2.93 to 1.14)	U = 1201.0, Z = − 0.16, *P* = .871	Z = − 3.47, *P* = .001
	Control(49)	107.56 ± 30.27	109.75 ± 28.75	2.19 ± 6.65			Z = − 2.12, *P* = .04

Values are presented as mean ± standard deviation (SD). Sleep quality was assessed using the Children’s Sleep Habits Questionnaire (CSHQ), with higher scores indicating poorer sleep quality. Parental stress was assessed using the Stress Response Inventory (SRI), with higher scores indicating greater stress. Change scores were calculated as post-intervention minus pre-intervention values. Within-group comparisons were performed using the Wilcoxon signed-rank test. Between-group comparisons of change scores were performed using the Mann–Whitney U test. CI = confidence interval

Within-group analyses showed small, non-significant reductions in sleep quality scores in both the intervention (Z = − 1.72, *P* = .08) and control groups (Z = − 1.41, *P* = .15). Parental stress scores increased significantly from baseline in both the intervention (Z = − 3.47, *P* = .001) and control groups (Z = − 2.12, *P* = .04).

Exploratory analyses of sleep subdomains demonstrated significant between-group differences favoring the intervention group in bedtime resistance (Z = − 2.47, *P* = .01), sleep anxiety (Z = − 2.69, *P* < .001), and parasomnias (Z = − 2.47, *P* = .01). At the parental stress subscale level, somatization scores differed significantly between groups following the intervention (Z = − 2.20, *P* = .02), with a smaller increase observed in the intervention group than in the control group. No significant between-group differences were identified for the remaining sleep subdomains (Table 4) or parental stress subscales (Table 5).

**Table 4:** Sleep subscale scores before and after the intervention and between-group comparisons

Subscale	Group(*n*)	Pre-Intervention (Mean ± SD)	Post-Intervention (Mean ± SD)	Within-group comparison*	Between-group comparison†
Sleep Resistance	Intervention(50)	12.09 ± 2.96	11.23 ± 2.39	Z = -3.74, *P* < .001	Pre: Z = -0.16, *P* = .86
	Control(49)	12.00 ± 3.05	12.58 ± 2.85	Z = -1.36, *P* = .17	Post: Z = -2.47, *P* = .01
Sleep Onset Delay	Intervention(50)	1.84 ± 0.73	1.80 ± 0.66	Z = -0.57, *P* = .56	Pre: Z = -0.25, *P* = .80
	Control(49)	1.89 ± 0.83	1.95 ± 0.74	Z = -0.36, *P* = .71	Post: Z = -1.03, *P* = .29
Sleep Duration	Intervention(50)	6.98 ± 1.51	6.72 ± 1.32	Z = -2.04, *P* = .04	Pre: Z = -1.42, *P* = .15
	Control(49)	7.41 ± 1.59	6.93 ± 1.77	Z = -1.22, *P* = .21	Post: Z = -0.56, *P* = .57
Sleep Anxiety	Intervention(50)	7.31 ± 2.43	7.03 ± 2.21	Z = -2.40, *P* = .01	Pre: Z = -1.56, *P* = .11
	Control(49)	7.77 ± 1.60	8.18 ± 1.94	Z = -1.65, *P* = .09	Post: Z = -2.69, *P* = .006
Night Awakenings	Intervention(50)	6.23 ± 1.28	6.07 ± 1.38	Z = -1.46, *P* = .14	Pre: Z = -1.14, *P* = .25
	Control(49)	6.60 ± 1.54	6.68 ± 1.44	Z = -0.41, *P* = .67	Post: Z = -1.78, *P* = .07
Parasomnias	Intervention(50)	18.03 ± 1.94	17.47 ± 2.14	Z = -2.37, *P* = .01	Pre: Z = -0.53, *P* = .59
	Control(49)	18.29 ± 1.78	18.54 ± 1.62	Z = -0.64, *P* = .51	Post: Z = -2.47, *P* = .01
Sleep-Disordered Breathing	Intervention(50)	7.31 ± 1.43	7.19 ± 1.16	Z = -0.83, *P* = .40	Pre: Z = -1.30, *P* = .19
	Control(49)	7.66 ± 1.38	7.50 ± 1.45	Z = -0.35, *P* = .72	Post: Z = -1.41, *P* = .15
Daytime Sleepiness	Intervention(50)	17.78 ± 2.54	17.49 ± 2.43	Z = -1.60, *P* = .10	Pre: Z = -0.37, *P* = .71
	Control(49)	18.14 ± 3.27	18.70 ± 3.14	Z = -1.27, *P* = .20	Post: Z = -1.83, *P* = .06

*Note: Wilcoxon signed-rank test was used for within-group comparisons (pre- vs. post-intervention). Mann-Whitney U test was used for between-group comparisons (intervention vs. control) at each time point. SD = Standard Deviation

**Table 5:** Parental Stress Subscale Scores Before and After the Intervention and Between-Group Comparisons

Subscale	Group(*n*)	Pre-Intervention (Mean ± SD)	Post-Intervention (Mean ± SD)	Within-group comparison*	Between-group comparison†
Tension	Intervention(50)	14.43 ± 5.26	14.84 ± 5.18	Z = -2.64, *P* = .008	Pre: Z = -0.31, *P* = .75
	Control(49)	14.14 ± 5.00	14.77 ± 4.94	Z = -1.75, *P* = .08	Post: Z = -0.09, *P* = .92
Aggression	Intervention(50)	7.13 ± 2.91	8.17 ± 3.23	Z = -4.69, *P* < .001	Pre: Z = -1.26, *P* = .20
	Control(49)	8.35 ± 4.11	8.20 ± 4.35	Z = -0.28, *P* = .77	Post: Z = -0.74, *P* = .45
Somatization	Intervention(50)	7.09 ± 2.55	7.76 ± 2.68	Z = -4.31, *P* < .001	Pre: Z = -1.36, *P* = .17
	Control(49)	8.02 ± 2.91	9.16 ± 2.93	Z = -4.12, *P* < .001	Post: Z = -2.20, *P* = .02
Anger	Intervention(50)	15.90 ± 6.00	16.17 ± 5.94	Z = -1.57, *P* = .11	Pre: Z = -1.04, *P* = .29
	Control(49)	14.70 ± 5.55	16.14 ± 5.73	Z = -2.21, *P* = .02	Post: Z = -0.01, *P* = .99
Depression	Intervention(50)	21.13 ± 8.02	21.37 ± 7.96	Z = -1.35, *P* = .17	Pre: Z = -0.66, *P* = .50
	Control(49)	20.04 ± 8.87	20.66 ± 8.69	Z = -3.47, *P* < .001	Post: Z = -0.47, *P* = .63
Fatigue	Intervention(50)	15.33 ± 5.00	15.43 ± 4.73	Z = -2.37, *P* = .01	Pre: Z = -0.75, *P* = .45
	Control(49)	14.66 ± 4.75	15.33 ± 4.66	Z = -0.64, *P* = .51	Post: Z = -0.05, *P* = .95
Hopelessness	Intervention(50)	20.49 ± 6.83	20.17 ± 6.71	Z = -0.45, *P* = .64	Pre: Z = -2.93, *P* = .003
	Control(49)	16.47 ± 7.01	17.91 ± 6.89	Z = -1.78, *P* = .07	Post: Z = -1.81, *P* = .06

Values are presented as mean ± standard deviation (SD). Parental stress subscales were measured using the Parenting Stress Index (PSI); higher scores indicate greater stress.^*^Within-group comparisons: Wilcoxon signed-rank test (pre-intervention vs. post-intervention).†Between-group comparisons: Mann-Whitney U test (intervention vs. control group) at each time point. *P* < .001 is reported for values below 0.001

Sensitivity analysis adjusting for sex confirmed that the baseline imbalance in sex distribution did not materially influence the findings. After adjustment, the intervention remained not significantly associated with post-intervention sleep quality (F(1,95) = 0.48, *P* = .49, partial η² = 0.005), and sex was not significantly associated with post-intervention parental stress scores (F(1,95) = 0.66, *P* = .42, partial η² = 0.007). Because analyses of sleep and stress subscales involved multiple comparisons, these findings should be interpreted as exploratory. 

At baseline, the Hopelessness subscale differed significantly between the intervention and control groups (*P* = .003), indicating a baseline imbalance for this specific subscale.

## Discussion

This randomized controlled trial evaluated the effects of a structured respiratory care education program for parents of children with neuromuscular diseases (NMDs) on children’s sleep quality and parental stress. Although no significant between-group differences were observed in overall sleep quality or total parental stress scores following the intervention, improvements were identified in selected sleep-related domains, including bedtime resistance, sleep anxiety, and parasomnias among children in the intervention group. In addition, differences in parental somatization scores were observed between groups after the intervention. These findings suggest that caregiver-focused respiratory education may improve selected aspects of nighttime functioning and caregiver well-being, although broader effects on overall sleep quality and parental stress remain uncertain. It should also be noted that a significant baseline imbalance was observed in the Hopelessness subscale; therefore, findings related to this specific outcome should be interpreted with caution.

The observed improvements in specific sleep domains are clinically plausible given the close relationship between respiratory dysfunction and nocturnal discomfort in children with NMDs. Our findings are partly consistent with previous studies showing that family-centered educational interventions can positively affect children’s behavioral and physiological outcomes, and that interventions such as muscle training and non-invasive ventilation improve nocturnal comfort and sleep continuity [20–22]. The improvements observed in sleep resistance and anxiety may stem from enhanced caregiver competence in managing nocturnal respiratory events, which reduces child arousal and anxiety during sleep. Similarly, reductions in parental somatization likely reflect decreased hypervigilance and increased confidence in caregiving, consistent with previous literature showing that caregiver education may reduce psychological burden in families of children with chronic illnesses [23–25] .

Notably, significant changes were not observed in overall sleep quality scores. Several factors may explain this finding. First, the relatively short duration of follow-up may have limited the ability to detect broader behavioral or physiologic changes. Improvements in respiratory care routines and caregiver confidence may require longer periods of implementation before measurable effects on overall sleep quality emerge. Second, participants in the present study were recruited from a subgroup of children with relatively elevated sleep disturbance scores identified during a prior descriptive assessment. Consequently, higher baseline symptom burden may have reduced the likelihood of observing substantial short-term changes in overall sleep outcomes.

With respect to parental stress, both groups demonstrated modest increases in total stress scores during the study period, and no significant between-group difference was observed in overall stress outcomes. These findings suggest that short-term educational interventions alone may be insufficient to substantially reduce the complex psychological burden associated with caregiving for children with progressive neuromuscular disorders. Caregiver stress in NMD is multifactorial and may be influenced by ongoing concerns regarding disease progression, respiratory complications, financial strain, and long-term caregiving demands. Although the overall between-group difference in stress was not statistically significant, the intervention group demonstrated a smaller post-intervention increase in total stress compared with controls. This pattern suggests that the education program may have mitigated, though not eliminated, the psychological strain of caregiving. It is plausible that parents who gained practical skills in respiratory care felt more competent in managing their child’s condition, buffering some of the emotional impact associated with chronic caregiving demands [26].

Nevertheless, differences in somatization scores between groups may indicate that respiratory care education influenced certain physical manifestations of stress. Parents who received structured education may have felt more prepared to manage respiratory symptoms and nighttime events, potentially reducing perceived physical strain associated with caregiving responsibilities. However, these findings should be interpreted cautiously because analyses of stress subscales were exploratory and involved multiple comparisons.

### Strengths and limitations

The present study has several strengths. To our knowledge, few randomized controlled studies have examined caregiver-focused respiratory education specifically among families of children with NMDs. Moreover, the achieved sample size is a notable strength, given that studies involving pediatric neuromuscular diseases are frequently underpowered because of recruitment challenges associated with the low prevalence of these conditions. The study also evaluated both child-centered and caregiver-centered outcomes, reflecting the multidimensional impact of chronic neuromuscular disorders. Furthermore, the online delivery format improved accessibility for families who may face mobility, transportation, or geographic barriers to in-person rehabilitation services. Another notable strength was the absence of participant attrition throughout the study. This high retention rate may be attributed to the short study duration, the convenience of the online educational program, the high level of parental motivation, and regular communication between the research team and participants, all of which likely facilitated continued engagement and study completion.

Several limitations should be acknowledged. First, the relatively short follow-up period limited the assessment of the long-term sustainability of the intervention effects. Future studies with longer follow-up periods are needed to determine whether these effects are maintained over time. Second, outcome measures relied on parent-reported questionnaires, which may be subject to reporting bias. Third, although randomization was performed, a baseline imbalance in child sex distribution was observed between groups; however, sensitivity analyses adjusting for sex yielded findings consistent with the primary results. Fourth, formal assessment of caregiver learning or competency following the educational sessions was not performed, limiting objective evaluation of intervention adherence and knowledge acquisition. Fifth, unmeasured contextual and environmental factors during the study period may have influenced parental stress levels independently of the intervention. Finally, analyses of the sleep and parental stress subscales were exploratory and should be interpreted with caution because of the increased risk of type I error associated with multiple comparisons.

Future studies should incorporate longer follow-up periods, objective sleep-related outcome measures, and more robust statistical approaches to clarify the long-term effects of caregiver respiratory education in pediatric NMD populations. Evaluating caregiver competency, adherence to respiratory care practices, and disease severity stratification may further help identify families most likely to benefit from such interventions.

This randomized controlled trial suggests that a structured respiratory care education program for parents of children with neuromuscular diseases may improve selected sleep-related domains, including bedtime resistance, sleep anxiety, and parasomnias. Although no significant improvements were observed in overall sleep quality or total parental stress scores, exploratory differences identified in specific sleep and stress subdomains suggest that caregiver-centered respiratory education may offer benefits in selected aspects of child sleep and caregiver well-being.

These findings support the feasibility and potential utility of incorporating respiratory care education into multidisciplinary pediatric neuromuscular rehabilitation programs, particularly in settings where families require additional support for home-based respiratory management. However, given the exploratory nature of the subscale analyses and the short follow-up period, the findings should be interpreted cautiously.

Further randomized studies with longer follow-up durations, objective outcome measures, and comprehensive assessment of caregiver competency are needed to clarify the long-term clinical effectiveness of caregiver-focused respiratory education interventions in children with neuromuscular diseases.

## Data Availability

The datasets generated and analyzed during the current study are available from the corresponding author on reasonable request.

## Abbreviations

NMDs: Neuromuscular diseases
SMA: Spinal muscular atrophy
CPR: Cardiopulmonary resuscitation
CSHQ: Children’s Sleep Habits Questionnaire
SRI: Stress Response Inventory
DMD: Duchenne muscular dystrophy
BMD: Becker muscular dystrophy
